# Case report: Early-onset osteoporosis in a patient carrying a novel heterozygous variant of the *WNT1* gene

**DOI:** 10.3389/fendo.2022.918682

**Published:** 2022-08-08

**Authors:** Maria Cristina Campopiano, Antonella Fogli, Angela Michelucci, Laura Mazoni, Antonella Longo, Simona Borsari, Elena Pardi, Elena Benelli, Chiara Sardella, Laura Pierotti, Elisa Dinoi, Claudio Marcocci, Filomena Cetani

**Affiliations:** ^1^ Department of Clinical and Experimental Medicine, Unit of Endocrinology, University of Pisa, Pisa, Italy; ^2^ Laboratory of Molecular Genetics, University Hospital of Pisa, Pisa, Italy; ^3^ Department of Biological Sciences and BioDiscovery Institute, University of North Texas, Denton, TX, United States; ^4^ Unit of Endocrinology, University Hospital of Pisa, Pisa, Italy

**Keywords:** Wnt signaling, fracture, teriparatide, osteogenesis imperfecta, *COL1A1*

## Abstract

The *WNT1* gene is crucial for bone development and homeostasis. Homozygous mutations in *WNT1* cause severe bone fragility known as osteogenesis imperfecta type XV. Moreover, heterozygous *WNT1* mutations have been found in adults with early-onset osteoporosis. We identified a 35 year-old Caucasian woman who experienced multiple vertebral fractures two months after her second pregnancy. There was no history of risk factors for secondary osteoporosis or family history of osteoporosis. Dual-energy X-ray absorptiometry confirmed a marked reduction of bone mineral density (BMD) at the lumbar spine (0.734 g/cm^2^, Z-score -2.8), femoral neck (0.48 g/cm^2^, Z-score -3.5), and total hip (0.589 g/cm^2^, Z-score -3.0). Blood tests excluded secondary causes of bone fragility. Genetic analysis revealed a heterozygous missense mutation (p.Leu370Val) in the *WNT1* gene. *Varsome* classified it as a variant of uncertain significance. However, the fact that the Leucine residue at position 370 is highly conserved among vertebrate species and the variant has a very low allelic frequency in the general population would exclude the possibility of a polymorphism. The patient was treated for two years with teriparatide therapy associated with calcium and vitamin D supplements. During the follow-up period she did not report further clinical fractures. After 24 months of teriparatide, BMD increased at lumbar spine (+14.6%), femoral neck (+8.3%) and total hip (+4.9%) compared to baseline. We confirm that the heterozygous *WNT1* mutation could cause a variable bone fragility and low turnover osteoporosis. We suggest that teriparatide is one of the most appropriate available therapies for this case.

## Introduction

Osteoporosis is a common skeletal disorder characterized by low bone mineral density (BMD), impaired bone quality, and increased risk of fragility fractures. It is typically age-related, but there might be concomitant causes, including genetic variants, diseases, and lifestyle habits. Conversely, early-onset osteoporosis is rather rare and might be due to single-gene mutations. Osteogenesis imperfecta (OI) is, by far, the most common monogenic form of bone fragility (1). It includes a wide spectrum of clinically and genetically heterogeneous diseases and its phenotype ranges from perinatal lethality to mild early-onset osteoporosis. Individuals with OI have bone fragility and deformity with or without growth deficiency, dentinogenesis imperfecta, hearing loss, blue sclerae, macrocephaly, and ligaments laxity (2–4). The most common types of OI are inherited in an autosomal dominant (AD) manner and mainly caused by heterozygous mutations of *COL1A1* or *COL1A2* genes (1). Conversely, up to 20% of OI is due to homozygous mutations in genes widely involved in bone homeostasis, namely Wnt Family Member 1 (*WNT1*) and low-density lipoprotein receptor-related protein 5 (LRP5), and inherited in an autosomal recessive (AR) manner (5–7).

The role of the *WNT* gene in bone formation and maintenance has been extensively studied since dysregulation of mediators of the Wnt/β-Catenin pathway has been associated with the onset of diseases with high or low bone-mass phenotypes (8–10). Particularly, *WNT1* promotes bone formation by binding to the LRP5-frizzled (FZD) receptor complex and activating the canonical Wnt/β-Catenin pathway (11).

Mutations of *WNT1* gene have been reported in patients with variable bone fragility disorders inherited either as AD or AR (12–17). Heterozygous *WNT1* mutations result in a mild reduction in Wnt/β-Catenin signaling (18), leading to mild bone disease, such as early-onset osteoporosis with multiple fractures, mainly affecting the spine; however, growth is not affected and extra-skeletal features are not common in these cases (19). Conversely, homozygous *WNT1* mutations display a severe phenotype, classified as OI type XV, characterized by reduced bone mass, multiple fractures, skeletal deformities, growth delay, dentinogenesis imperfecta, hearing loss, blue sclerae, and psychomotor disability (12–14, 19).

To date, it is unclear why homozygous and heterozygous *WNT1* mutations may cause different bone fragility pictures, even in the same kindred. Thus, the effect of both heterozygous and homozygous *WNT1* mutations and the relationship between *WNT1* variants and environmental factors requires further elucidation. Furthermore, the skeletal effects of *WNT1* mutations and the appropriate treatment remain a matter of concern. To date, the first-line treatment of OI is represented by bisphosphonates, regardless of its genetic profile.

In this report, we describe a 35 year-old Caucasian woman affected by early-onset osteoporosis with several vertebral fractures occurring during the second *puerperium* period. A detailed clinical evaluation suggested a genetic cause of the disease, which was confirmed by the identification of a variant of the *WNT1* gene.

## Case description

**Figure 1:**
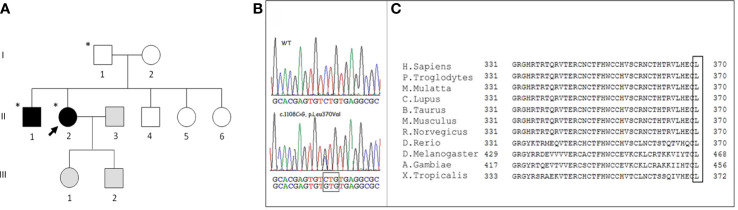
**(A)** Family pedigree. Squares represent men, circles women, black symbols affected family members, white symbols unaffected members, grey symbols members not evaluated; the asterisk identifies subjects carrying the *WNT1* p. Leu370Val variant. The arrow identifies the proband. **(B)** Representative Sanger sequencing results of a healthy subject and the proband: the sequencing chromatogram of the index patient indicates a heterozygous *WNT1* c.1108C>G mutation (nomenclature according to National Center for Biotechnology Information Reference Sequence: NM_005430). **(C)** Alignment of Wnt1 protein in different vertebrate species. The highly conserved last amino acid (L) of Wnt1 is highlighted in the box.

The proband, a 35 year -old Caucasian woman (**Figure 1**, II.2), was referred to our outpatient clinic in June 2019 for evaluation of early-onset osteoporosis. Her past medical history was unremarkable until the second *puerperium*. Growth and psychomotor development were regular, without events in the neonatal period and infancy. She had never taken corticosteroid, anti-epileptic, or serotoninergic medications. The patient was a non-smoker and had no alcohol abuse. At the age of 30 years, she had an uneventful first pregnancy with breastfeeding.

**Figure 2:**
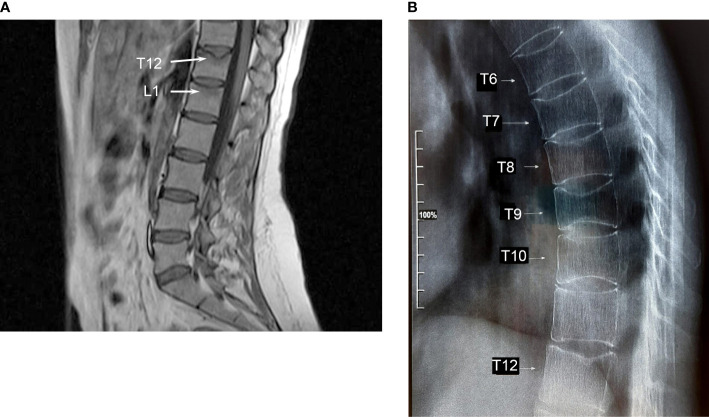
**(A)** Magnetic resonance imaging of the lumbar spine revealing the presence of vertebral fractures of T12 and L1. Peri-lesion edema of both vertebrae indicating recent fractures is shown (arrows). **(B)** X-ray of the thoracic spine shows vertebral fractures from T6 to T9.

In February 2018, two months after the second delivery, while breastfeeding, she experienced an acute and spontaneous thoracic-lumbar back pain. Magnetic resonance imaging (MRI) of the lumbar spine revealed vertebral fractures of the T12 and collapse to wedge-shape of L1, without posterior height alteration. Moreover, the MRI signal suggested the presence of peri-lesion edema, as it was hypointense in T1 and hyperintense in STIR sequences indicating the recent nature of the vertebral fractures (**Figure 2**). At this time, no further evaluation was performed. She wore a back brace for three months and started cholecalciferol supplements (monthly 50000 U.I.). No further advice was given to the patient and she continued breastfeeding. In January 2019, X-ray of the whole spine showed additional vertebral fractures from T6 to T9 (**Figure 2**). Dual-energy X-ray absorptiometry (DXA) showed a marked reduction of the BMD at the lumbar spine (0.734 g/cm^2^, Z-score -2.8), femoral neck (0.48 g/cm^2^, Z-score -3.5), and total hip (0.589 g/cm^2^, Z-score -3.0).

At the time of our evaluation, the patient was in good health. Physical examination showed moderate kyphosis, without appendicular skeleton abnormalities and signs of ligaments laxity. Blue sclerae and dental abnormalities were not evident.

The estimated daily calcium intake, using a self-administered questionnaire, was 500 mg (20).

Laboratory tests of bone metabolism were unremarkable although bone formation markers, e.g., bone-specific alkaline phosphatase (BALP), osteocalcin, and the serum bone resorption marker cross-linked C-telopeptide of type I collagen (S-CTX) were in the low-normal range (**Table 1**). Unfortunately, baseline value of serum amino-terminal propeptide of type 1 collagen (P1NP) was not available. Serum 25-hydrovitamin D was 30 ng/mL. Additional blood tests were performed to rule out secondary causes of osteoporosis, which could be excluded (data not shown).

**Table 1:** Biochemical parameters at baseline and during teriparatide treatment.

Parameters	Normal range	Baseline	6 months	12 months	18 months	24 months
Total serum calcium	8.6-10.2 mg/dL	9.2	9.6	8.8	9.1	8.6
Phosphate	2.5-4.5 mg/dL	3.3	3.9	3.2	2.7	3.6
P1NP	27-127 µg/L	NA	36	29	26	44
BALP	4.7-27.1 µg/L	7.1	9.6	11.7	NA	NA
Osteocalcin	6.8-34.0 ng/mL	6.0	18.6	22.4	11.5	8.0
S-CTX	0.034-0.635 µg/L	0.099	0.102	0.171	0.184	0.222

BALP, bone specific alkaline phosphatase; S-CTX, serum carboxy-terminal collagen crosslinks; P1NP, procollagen type 1 amino-terminal pro-peptide; NA, not available.

The finding of early-onset fractures led us to suspect a genetic cause. Family history was negative for osteoporosis and fragility fractures. All family members (**Figure 1**) were in good health except the younger sister (**Figure 1**, II.6) who had a congenital myopathy associated with an unspecified cardiopathy. No history of consanguinity was present.

Blood samples were obtained between 8 and 9 a.m. after an overnight fast. Serum calcium, creatinine, and phosphate were determined using standard methods. Plasma PTH was measured by a third-generation assay (DiaSorin LIAISON 1-84 PTH chemiluminescent immunoassay) and serum 25-hydroxyvitamin D (25[OH]D) was measured by a chemiluminescent immunoassay (IDS-iSYS); Bone-specific alkaline phosphatase (B-ALP) by immunoenzymatic assay (OCTEIA Ostase BAP; IDS Ltd., Boldon, Tyne & Wear, UK), and serum N-MID osteocalcin (IDS Ltd., Boldon, UK), serum P1NP and S-CTX (Nordic Bioscience Diagnostics A/S, Herlev, Denmark) by ELISA. The reference ranges are reported in **Table 1**.

BMD was measured by DXA using Hologic QDR-4500 (Hologic Inc., Waltham, MA, USA) at the lumbar spine in posterior-anterior projection (L1–L4), femoral sites, and total femur. The coefficients of variations were 1.1% at lumbar spine and 1.2% at femoral neck.

The evaluation of vertebral fractures was made by vertebral fracture assessment (VFA) using DXA images (two lateral scans of the vertebrae from T4 to L4). The accuracy of VFA had been validated in a previous study (21).

In June 2019, the patient started treatment with teriparatide 20 mcg daily, calcium citrate 500 mg daily and continued cholecalciferol supplement. In addition, a performing resistance training was also recommended.

The patient was followed every six months at our outpatient clinic. Of note, both formation and resorption bone markers increased during treatment (**Table 1**).

The patient showed significant increases in serum bone formation markers BALP and osteocalcin during the first 6 to 12 months of treatment (35%–65% and 210%–273%, respectively) (**Table 1**). Osteocalcin stayed above the baseline throughout the 24 months of intervention. Unfortunately, baseline value was not available for P1NP. Serum bone resorption marker CTX markedly increased from 3% during the first 6 months to 124% after 24 months of treatment (**Table 1**).

During the follow-up the patient was in good health and did not report bone pain or further clinical fractures. The treatment was well tolerated and no adverse events occurred.

After 24 months of teriparatide treatment, BMD increased at lumbar spine, femoral neck, and total hip compared to baseline (lumbar spine: 0.841 g/cm^2^ vs. 0.734, + 14.6%; femoral neck 0.52 g/cm^2^ vs. 0.48, + 8.3%; total hip 0.618 g/cm^2^ vs. 0.589, + 4.9%).

At 12 and 24 months of teriparatide treatment, VFA did not show additional vertebral fractures.

The method of genetic analysis is described in the “Materials and Methods” section. Targeted NGS was carried out to screen the causative genes of OI or bone fragility disease for the proband. In total, 40 variants, including 30 exon and 10 intron variants, were identified. After filtering, one candidate heterozygous missense variant, c.1108C>G (p.Leu370Val) (rs897040674 dbSNP), in the *WNT1* gene (NM_005430) was found (**Figure 1**). The variant was confirmed in the DNA of the patient by Sanger sequencing. Finally, by investigating her relatives by Sanger, the same genetic alteration was found both in the proband father (**Figure 1**, I.1) and in the older brother (**Figure 1**, II.1), while its presence in the mother, both sisters and the younger brother was excluded. The genetic analysis was not performed in the proband children because of the lack of consent.

This variant was not present in ClinVar, HGMD, and LOVD 3.0 databases.

The verdict of VarSome analysis (22) classified the variant as a variant of uncertain significance (VUS). According to ACMG guidelines, the variant was reported as a VUS due to conflicting pathogenic and benign lines of evidence, detailed as follows: (i) PM1: located in a mutational hot spot and/or critical and well-established functional domain without benign variation; (ii) PM2: absent from controls in Exome Sequencing Project, 1000 Genomes Project, or Exome Aggregation Consortium; (iii) BP4: no impact on gene or gene product suggested by multiple lines of computational evidence. Six tools classified the variant as damaging, disease causing, or deleterious. Conversely, seven tools suggested, with a low strength, that the variant had no impact on gene product (**Table 2**). In GnomAD (available at https://gnomad.broadinstitute.org/), the allelic frequency of this variant is very low (0.0000311), confirming the classification of the variant as a VUS. Of note, most missense variants of *WNT1* are reported as a common cause of disease, with a low rate of benign missense variants.

**Table 2:** Effect of the *WNT1* p.Leu370Val variant using several *in silico* methods.

Tool	Prediction	Pathogenicity classifier approach
MutationTaster	Disease causing	Based on evolutionary conservation, splice-site, mRNA, protein and regulatory features
MutationAssessor	Medium	Predicts the functional impact of variation in proteins through sequence conservation of protein homologs
DEOGEN2	Damaging	Incorporates heterogeneous information about the molecular effects of the variants, the domains involved, the relevance of the gene and the interactions in which it participates
FATHMM-MKL	Damaging	Predicts noncoding effects by integrating functional annotation information from the ENCODE
M-CAP	Deleterious	Based on an hybrid ensemble score
CADD	Deleterious	Based on genomic features derived from surrounding sequence contest, gene model annotations, evolutionary constraint, epigenetic measurements and functional predictions
BayesDel_addAF	Benign	It is a deleteriousness meta-score
DANN	Benign	A deep learning approach
EIGEN	Benign	A spectral approach integrating functional genomic annotations
LIST-S2	Tolerated	Predicts the deleteriousness by aligning the query sequence to all protein sequences in the UniProt Swiss-Prot/TrEMBL database and estimating the potential deleteriousness based on taxonomy distance of species
MVP	Benign	Predicts pathogenicity of missense variants by deep learning
PrimateAI	Tolerated	It is a deep residual neural network for classifying the pathogenicity of missense mutations
SIFT	Tolerated	Based on sequence homology derived from closely related sequences collected through PSI-BLAST

Using NCBI, we found that the *WNT1* 370 Leucine residue was highly conserved among vertebrate species (**Figure 1**).

### Densitometric and radiological features of the relatives carrying the variant

The proband father DXA at lumbar spine and femoral neck showed a BMD of 1.066 g/cm^2^ with T-score of -1.4 and 0.984 g/cm^2^ with T-score of -0.96, respectively. The X-ray of the whole spine excluded vertebral fractures.

The older proband brother DXA at lumbar spine and femoral neck showed a BMD of 0,790 g/cm^2^ with Z-score of -2,6 and 0.613 g/cm^2^ with Z-score -1.9, respectively. The X-ray of the whole spine displayed a severe collapse to wedge-shape of T10. Of note, no history of trauma was reported by the subject.

## Materials and methods

### Genetic analysis

DNA samples were collected from peripheral blood and extracted using the QIA symphony automatic instrument (QIAGEN, Germany). For the Next Generation Sequencing (NGS) analysis, we developed a panel using a custom-designed Agilent SureSelect QXT target sample library preparation and capture method (Agilent Technologies, USA). We performed parallel high-throughput sequencing analysis of the coding exons and flanking regions of 19 genes associated with osteogenesis imperfecta or with bone fragility (*COL1A1* (NM_00088), *COL1A2* (NM_00089), *IFITM5* (NM_025092), *BMP1* (NM_006129), *CRTAP* (NM_006371), *FKBP10* (NM_021939), *LEPRE1* (NM_001243246), *PPIB* (NM_024798), *SERPINH1* (NM_001235), *WNT1* (NM_005430), *SERPINF1* (NM_002615) and *SP7* (NM_001173467), *ANO5* (NM_213599), *CASR* (NM_000388), *CLCN5* (NM_001127898), *GNAS* (NM_000516), *LRP5* (NM_002335), *MMP2* (NM_004530), *TNFRSF11A* (NM_003839)). A total of 10 ng DNA/sample was used for the target enrichment step. For quantification of the samples and to verify the length of the library, we used the Qubit dsDNA HS Assay kit in a Qubit instrument (Invitrogen, USA) and the High Sensitivity DNA kit in an Agilent 2100 Bioanalyzer (Agilent Technologies, USA). The captured libraries were sequenced with a MiSeq Sequencer (Illumina, USA). Read alignment, variant calling, and annotation were performed with the Agilent SureCall software (Agilent Technologies, USA). Finally, the sequencing coverage of each exon was analyzed in detail using IGV tool (Integrative Genomics Viewer, Broad Institute and the Regents of the University of California, USA), to reduce the risk of incorrect results and for the detection of deletions.

### Variant classification

To assess the pathogenicity of the variant of interest, we used VarSome (available at https://varsome.com), which estimates the impact of mutations on protein structure and function. It is based on an accurate analysis of NGS data from different databases, including UniProt Variants, dbscSNV, DANN SNVs, gnomAD, and ClinVar. Pathogenicity classification of sequence variants based on the American College of Medical Genetics and Genomics (ACMG) guidelines (23) was automatically done in VarSome. According to the guidelines, the variant of interest is classified as pathogenic, likely pathogenic, benign, likely benign, or uncertain significance.

### Alignment

Protein alignment was performed using the National Center for Biotechnology Information (https://www.ncbi.nlm.nih.gov/). Among vertebrate species, several structurally related genes of *WNT1* were selected and compared.

## Discussion

It is well-known that pregnancy and breastfeeding may cause a transient reduction of bone mass *per se.* In both phases, placenta and breast produce PTH-related peptide to overcome the increased request of calcium, phosphate, and magnesium (24). Vertebral fractures rarely occur during the third trimester of pregnancy or breastfeeding and environmental factors, such as exercise habits, might increase the risk of fractures (25). Since 1993, the relevance of the genetic background underlying osteoporosis during pregnancy or lactation has been suggested by Dunne et al. (26), who observed a higher prevalence of family history for osteoporotic fractures during pregnancy in women with fractures compared to those without. In line with these observations, Butscheidt et al. (27) confirmed that mutations in genes involved in bone homeostasis were present in 3/7 (43%) of women having fractures during pregnancy.

**Figure 3:**
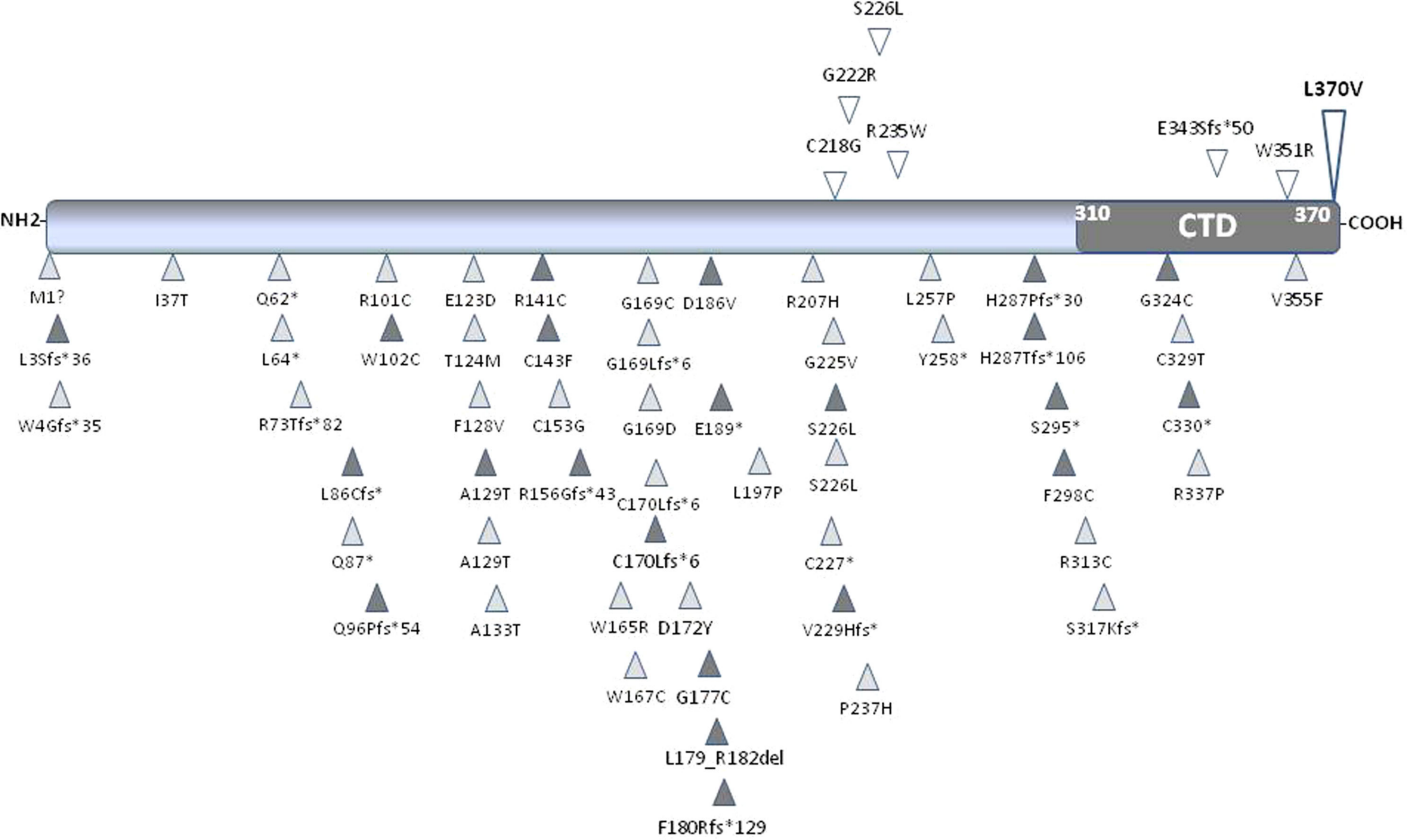
Schematic representation of Wnt1 protein. The carboxyl-terminal domain (CTD) is shown in dark grey. All the published missense, nonsense and frameshift *WNT1* variants associated with early-onset osteoporosis (upper part of the protein) and osteogenesis imperfecta (lower part of the protein) are shown and positioned in scale. The variant identified in this study is shown in bold. Empty symbols indicate heterozygous variants, dark-grey filled symbols indicate homozygous variants, and light-grey filled symbols indicate compound heterozygous variants.

Given the young age of our proband and the absence of secondary causes of osteoporosis, we hypothesized a genetic origin for the bone disease which was indeed confirmed by the finding of a heterozygous missense variant of the *WNT1* gene. *WNT1* encodes Wnt protein involved in Wnt/β-Catenin pathway which promotes osteoblastogenesis (18). *WNT1* pathogenic variants impair β-Catenin translocation affecting osteoblast function and peak bone mass (28, 29). *WNT1* pathogenic variants described so far (**Figure 3**) are associated with different pictures of bone fragility according to whether the variant was heterozygous or homozygous (13–17, 30–46). In fact, patients carrying heterozygous variants exhibit normal growth, reduced bone mass or early-onset osteoporosis and fractures without appendicular deformities (13–15, 42) (**Table 3**), whereas those carrying homozygous variants display a life-threatening phenotype due to severe bone deformities (12–14, 19). Notably, different Wnt proteins are expressed in bone, but Wnt1 is mainly responsible for bone homeostasis during skeletal development, bone mass accrual, and microarchitectural regulation (48, 49). It has been speculated that *WNT1* mutations may reduce Wnt1 ability to induce Wnt/β-Catenin pathway in varying degrees according to the homo or heterozygous nature (12, 43, 47), suggesting the presence of a phenotypic threshold effect. Indeed, our proband showed a clinical picture characterized by early-onset osteoporosis associated with multiple fragility vertebral fractures. The proband’s brother, who carries the same variant, also had a radiological vertebral fracture in the absence of clinical history of trauma. Conversely, the proband’s father, who also carries the variant, had a normal BMD and no clinical and radiological vertebral fractures. As a matter of fact, literature data confirm that individuals harboring heterozygous *WNT1* missense variants have a different clinical phenotype, ranging from normal or slightly reduced bone mass to early onset osteoporosis (13, 14, 37, 38, 40, 45).

**Table 3:** Clinical and genetic data of germline heterozygous missense *WNT1* variants reported in the literature.

*WNT1* mutation	p.C218G	p.G222R	p.S226L	p.E343Sfs*50	p.R235W	p.W351R
**N. of carrier**	24	2	2	1	4	4
**Age at diagnosis** **Mean [Range]**	34 years [10-68]^a^	9 and 59 years	56, 62 years	35 years	42 years [6-74]	39 years [10-80]
**Fragility fractures** **Yes/No** **[n]**	Yes	Yes^b,c^	No	Yes	Yes^b^	Yes
**Bone deformities**	Yes	No	No	No	No	No
**Blue sclerae**	Yes	No	No	NA	No	No
**Dentinogenesis imperfecta**	No	No	No	NA	No	No
**Hearing loss**	No	No	No	NA	No	No
**Joints hyperlaxity**	No	No	No	NA	No	Yes
**Short stature**	Yes	No	No	Yes	No	No
**CNS impairment**	No	No	No	NA	No	No
**Bone turnover**	Low-to-normal^d^	NA	NA	NA	Low^e^	NA
**BMD**	Low-to-normal^f^	Low^f^	Low-to-normal^f^	Low^f^	Low^g^	Low^f^
**Authors**	Laine et al., 2013 (14), Makitie et al., 2016 (16)^i^	Ang et al., 2018 (40)	Chen et al., 2020^l^ (47)	Caparros-Martin et al., 2017 (33)	Keupp et al., 2013 (13)	Alhamdi et al., 2018 (15)

HET, Heterozygous; HOM, homozygous; CNS, central nervous system; BMD, bone mineral density.^a^Mean age and range refers to 18/24 carriers^; b^asymptomatic vertebral fractures; ^c^during childhood or adolescence; ^d^histomorphometry; ^e^biomarkers; BMD evaluated at ^f^DXA, ^g^qCT, ^h^X-ray; ^i^Makitie et al., in addition to 10 previously reported mutation-positive subjects, screened further members of the same family firstly described by Laine et al. altogether with a second seemingly unrelated family; ^l^Chen et al. reported WNT1 missense variant associated to other genes mutation.

The *WNT1* p.Leu370Val variant has never been reported in mutation databases. Of interest, the alignment showed that Wnt1 370 Leucine residue is highly conserved among vertebrate species. 370 Leucine is the last residue located in the C-terminal domain (CTD, 310-370 aa) of the Wnt1 protein. A structural model of the protein based on the crystal structure of the highly homologous human Wnt3 protein (pdb: 6ahy) used as a template (50), suggests that Leu370 is located at the end of a beta-strand that is part of the C-terminal cysteine-rich domain. In this model, the Leu370 side chain is sandwiched between two arginines, Arg332 and Arg335. Since the leucine to valine mutation results into a shorter side chain, we can hypothesize that the residue might have a role in stabilizing the beta-strand or the linker region between the N- and C-terminal domain.

Moreover, six *in silico* prediction tools defined the *WNT1* p.Leu370Val variant as potentially damaging, especially when the allelic frequency and the residue conservation among species were considered in the algorithm, as well as the high prevalence of disease reported in patients carrying missense variants. Finally, the allelic frequency of this variant in the general population is very low (0.00003%), confirming the classification of the variant as a VUS.

Other missense variants closest to residue 370 are Trp351Arg and Val355Phe, both located in the CTD of Wnt1 protein. The former has been reported in a family with early-onset osteoporosis (15) and the latter in a 35 year-old woman described with short stature, vertebral fractures, and early-onset osteoporosis. The same mutation was also detected in the heterozygous state in her mother who only presented osteoporosis after menopause (33). Of note, *in vitro* studies observed that Trp351Arg had a significant reduced capacity to activate canonical Wnt signaling compared with the wild type (15). Conversely, Val355Phe is located in the ‘index finger’ of the Wnt1 protein. This amino acid residue seems to interact with three amino acids on the surface of the Frizzled receptor. The substitution of a valine by the larger side chain of the phenylalanine is likely to disrupt the structure of the finger region due to the clash with the residue Phe349. As suggested by the authors, this change would destabilize the interaction between Wnt1 and Frizzled receptor (33).

The first line treatment of OI is represented by bisphosphonates, however low turnover osteoporosis rarely takes advantage from anti-resorptive drugs (13). Indeed, the proband had a significant improvement of BMD, especially at the lumbar spine and no additional clinical and radiological fragility fractures after a 24-month treatment of teriparatide. The treatment induced a prompt increase of bone apposition markers. such as osteocalcin and BALP during the first 12 months. Osteocalcin peaked at 273% above the baseline at 12 months and then declined at 18 and 24 months (92% and 33%, respectively). Bone resorption markers such as serum CTX increased to a lesser extent up to 12 months (3% and 73% after 6 and 12 months, respectively), but continued to rise up 124% over the baseline during 24 months of treatment. Of note, teriparatide-treated patients with postmenopausal and idiopathic premenopausal osteoporosis showed a similar behavior of bone formation markers that peaked at 6 months but in contrast with our results, bone resorption markers tended to decline to the baseline after 6 months (51–53).

The results of our study show that teriparatide causes an increase of bone mass and improvement in skeletal architecture and is effective in this setting (54). In keeping with these findings, three patients carrying the heterozygous *WNT1* c.652T>G variant also exhibited an increase in both apposition and resorption markers; a mean increase of lumbar (+5.3%) and femoral neck (+2%) BMD compared to the baseline after a 24-month teriparatide treatment (54). Moreover, histomorphometry of the iliac crest biopsies of all three patients showed an increase in osteoid surface (mean +349%), osteoid volume (mean +749%), osteoid thickness (mean +57%), and a decrease of the eroded surface (-72%) (54). Unfortunately, we were not able to have such data in our proband since the patient did not give her consent to perform a bone biopsy. In Italy, teriparatide is dispensed by the National Health System for two years only. The ideal therapeutic target in patients carrying *WNT1* mutation might be sclerostin. This protein is codified by the *SOST* gene (17q.21.31) and binds to the co-receptors LRP-5/-6 to inhibit Wnt/β-Catenin pathway. The efficacy of anti-sclerostin antibodies was demonstrated in a mouse model with homozygous *WNT1* deletion (55). The question of whether anti-sclerostin antibodies might be effective in the treatment of OI or early-onset osteoporosis caused by heterozygous *WNT1* mutations remains to be elucidated.

In conclusion, the results of our study suggest a potential role of heterozygous *WNT1* variants in the pathogenesis of early-onset osteoporosis. In clinical practice, we underscore the need for careful clinical, biochemical and radiological evaluation to detect underlying secondary causes of the disease in patients with early-onset osteoporosis. Moreover, the genetic evaluation is also of great importance to exclude the known monogenic forms of osteoporosis. Finally, the results of genetic testing will also affect the patient’s medical care and follow-up. We suggest that teriparatide is one of the most appropriate available therapies for such cases.

## Data availability statement

The original contributions presented in the study are publicly available. This data can be found here: https://www.ncbi.nlm.nih.gov/genbank/, STUDY: PRJNA824693 SAMPLE: 191424 (SAMN27412350) EXPERIMENT: 3144231 (SRX14785074) RUN: 191424.bam (SRR18683929).

## Ethics statement

The studies involving human participants were reviewed and approved by University Hospital of Pisa Ethics Committee. The patients/participants provided their written informed consent to participate in this study. Written informed consent was obtained from the individual(s) for the publication of any potentially identifiable images or data included in this article.

## Author contributions

MCC, CM and FC contributed to the study conception, design and wrote the first draft of the manuscript. AF and AM performed the genetic analysis. AL contributed to the evaluation of the impact of the variant. SB, EP, CS, LP and ED collected all the data and revised the manuscript. All authors read and approved the final manuscript. FC is a co-coordinator of the Mineral and Bone Club of the Italian Society of Endocrinology (part of research projects).

## Funding

This work was supported by Fondi di Ateneo (CM)

## Acknowledgments

We wish to thank the members of the family who graciously agreed to collaborate in the study.

## Conflict of interest

The authors declare that the research was conducted in the absence of any commercial or financial relationships that could be construed as a potential conflict of interest.

## Publisher’s note

All claims expressed in this article are solely those of the authors and do not necessarily represent those of their affiliated organizations, or those of the publisher, the editors and the reviewers. Any product that may be evaluated in this article, or claim that may be made by its manufacturer, is not guaranteed or endorsed by the publisher.
